# NiO/Carbon Aerogel Microspheres with Plum-Pudding Structure as Anode Materials for Lithium Ion Batteries

**DOI:** 10.3390/ma13102363

**Published:** 2020-05-21

**Authors:** Renqing Guo, Xiaohua Huang, Yan Lin, Yiqi Cao

**Affiliations:** Department of Materials Engineering, Taizhou University, Taizhou 318000, China; guorenq@tzc.edu.cn (R.G.); linyan@tzc.edu.cn (Y.L.); caoyqtzc@163.com (Y.C.)

**Keywords:** nickel oxide, carbon aerogel, microsphere, lithium-ion battery

## Abstract

To enhance the electrochemical performance of nickel oxide as anode materials for lithium ion batteries, NiO/carbon aerogel microspheres with a plum-pudding structure were designed and prepared by a sol-gel technique followed by two calcination processes under different atmospheres. Carbon aerogel microspheres (pudding) can act as a buffering and conductive matrix to enhance the structural stability and conductivity of the embedded NiO particles (plums), which are quite advantageous to the cycling performance and rate capability. Consequently, NiO/carbon aerogel microspheres with a plum-pudding structure deliver an initial charge capacity of 808 mAh g^−1^ and a reversible capacity retention of 85% after 100 cycles. The enhancement in electrochemical performance relative to pure NiO microspheres suggests that the design of a plum-pudding structure is quite effective.

## 1. Introduction

Transition-metal oxides, especially iron group metal oxides, are developing as anode materials for lithium-ion batteries because of their high capacity, low cost, easy preparation, and environmentally friendly nature. They deliver high capacities ranging from 700 to 1000 mAh g^−1^ based on the mechanism of reversible conversion reaction [1,2]. The reaction involves large volume expansion and causes particle pulverization, which further leads to the separation of materials from the electrode, and the generation of new surfaces to produce more solid electrolyte interface (SEI). Therefore, iron group metal oxide anode materials often exhibit poor actual electrochemical performance including fast capacity fading and low coulombic efficiency, making it currently difficult for the materials to meet practical applications.

Fabricating nanostructured composites is the most frequent method to enhance the electrochemical performance of energy conversion and storage materials [3,4,5,6,7,8]. The nanostructures can increase the reaction interface and shorten the charge transport distance, thus reducing electrode polarization. Forming composites usually produces better conductivity and structural stability. During the past decade, various nanostructures have been designed for active materials such as low-dimensional [9,10], porous [11,12], spherical [13,14,15,16], hollow [17,18], core/shell [19], yolk/shell [20,21], and so on, meanwhile, conductive ductile matrices such as metal [22,23] and/or carbon [24,25,26] have been introduced to fabricate nanocomposites. It is known from these examples that many novel-structured nanocomposites show significantly enhanced electrochemical performance. Therefore, rational designing of the structure and component of electrode materials is extremely crucial to the improvement of electrochemical performance.

In this present work, NiO/carbon aerogel microspheres with a plum-pudding structure were well designed, carefully fabricated, and tested in detail as anode materials for lithium-ion batteries. The plum-pudding structure significantly enhanced the reversible capacity, cycling stability, and rate capability.

## 2. Materials and Methods

### 2.1. Sample Preparation

**Figure 1:**
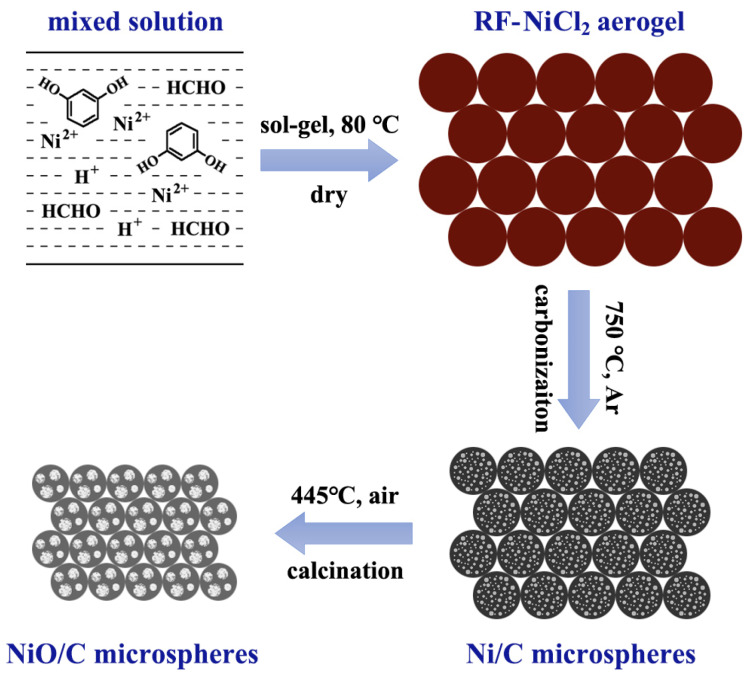
Schematic diagram of the material preparation process.

The sample was prepared by a sol-gel method using nickel salt and resorcinol-formaldehyde (RF) organics as raw materials. Microsphere particles can be obtained by controlling RF monomer concentration in the initial solution [27], and metals or metal oxides can be produced after the subsequent carbothermal reduction or air oxidation processes, respectively [28]. The preparation proceeded in three steps, as shown in Figure 1. The first step was the preparation of a NiCl_2_/RF aerogel precursor. In a 200 mL beaker, 10 mmol nickel chloride, 60 mmol resorcinol, 9 mL formaldehyde (37 wt%), and 0.5 mL hydrochloric acid (37 wt%) were dissolved in 80 mL deionized water to form a solution. The beaker was covered by plastic wrap and placed in a water bath maintained at 80 °C for 3 h. The resulting gel was dried at 80 °C for several days in air until the weight became stable. The second step was the carbonization of the NiCl_2_/RF aerogel. In a quartz-tube furnace, the NiCl_2_/RF aerogel was carbonized at 750 °C for 3 h under flowing argon and a Ni/C aerogel was produced. The last step was the oxidation of the Ni/C aerogel. In a box furnace, the Ni/C aerogel was calcined in air at 445 °C for 16 h in air using a heating rate of 2.5 °C min^−1^ as well as a free cooling rate, and the final product (i.e., NiO/C aerogel microspheres) was obtained.

Pure NiO microspheres were also prepared for a comparison of electrochemical performance by calcining the Ni/C aerogel in air at 445 °C for 24 h under the same other conditions.

### 2.2. Material Characterization

The samples were characterized by means of X-ray diffraction (XRD, D8 advance, Bruker, Karlsruhe, Germany) and scanning electron microscopy (SEM, S-4800, Hitachi, Tokyo, Japan, equipped with energy dispersive spectrometer (EDS)).

### 2.3. Electrochemical Measurements

For electrochemical tests, a slurry was prepared to fabricate working electrodes. The slurry consisted of active materials, acetylene black, and polyvinylidene fluoride (PVDF) (dissolved in N-methyl-pyrrolidone (NMP)) in a mass ratio of 8:1:1. Working electrodes, in which the areal density of the electrode material was about 0.9 mg cm^−2^, were fabricated by spreading the slurry on copper foil, drying in vacuum, and being punched into wafers. In an argon-filled glove box, the working electrode was assembled into a 2025 coin-type half-cell, together with a lithium wafer as a counter electrode, and 1 M LiPF_6_ dissolved in a mixed solvent of ethylene carbonate (EC), dimethyl carbonate (DMC), and ethyl methyl carbonate (EMC) with a volume ratio of 1:1:1 as the electrolyte.

Electrochemical investigations were performed at a constant temperature of 25 °C. The cells were subjected to galvanostatic discharge and charge tests on a program-controlled battery test system (CT2001A, LAND, Wuhan, China) at 100 mA g^−1^ between 0.02 and 3 V for 100 cycles. Subsequently, the current density was changed to 200, 500, 1000, 2000, and 100 mA g^−1^ in sequence for the evaluation of rate capability. The specific capacity was calculated from the mass of NiO/C composite in the electrode including NiO and carbon aerogel. Cyclic voltammetry (CV) analysis of the electrodes was performed on an electrochemical workstation (PGSTAT302N, Metrohm Autolab, Utrecht, The Netherlands) between 0 and 3 V using a scan rate of 0.1 mV s^−1^. Electrochemical impedance spectroscopy (EIS) was also carried out on the electrochemical workstation over a frequency range of 0.01 to 100 kHz using a small voltage signal of 5 mV in amplitude.

## 3. Results and Discussion

**Figure 2:**
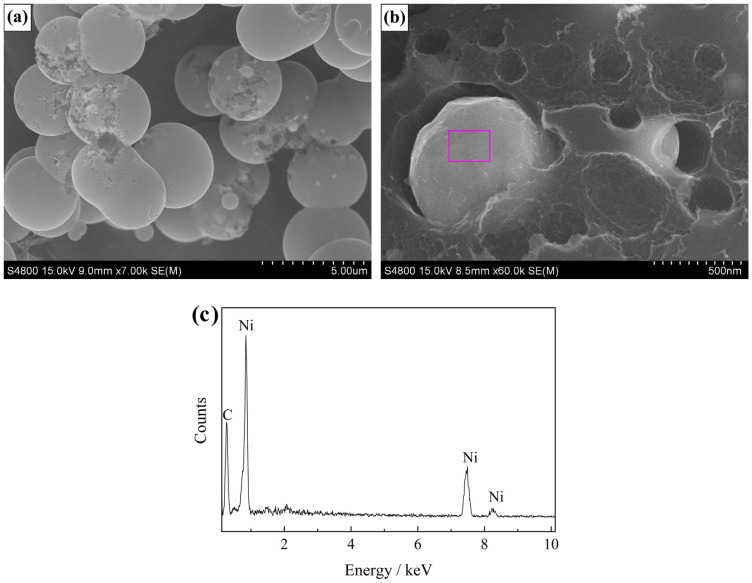
Scanning electron microscopy (SEM) characterization results of the Ni/C aerogel microspheres obtained after the carbonization process in argon: (**a**) low magnification image, (**b**) high magnification image, and (**c**) the corresponding energy dispersive spectrum (EDS).

Figure 2a presents the SEM image of Ni/C aerogel. The aerogel was constructed by interconnected microspheres, and most of them were quite regular in shape. They were close in diameter and the average value was about 4 μm. From some broken microspheres, some particles could be seen, and according to the magnified image (Figure 2b), the particles were sharp-edged and embedded inside the carbon microsphere matrix. The EDS pattern (Figure 2c) recorded on the particle area showed elements of Ni and C, and no other elements were identified, indicative of complete carbonization and reduction.

**Figure 3:**
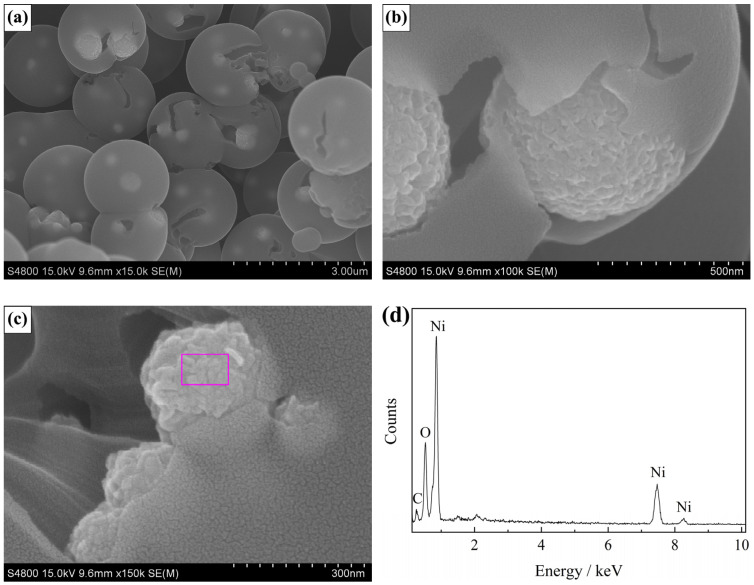
SEM characterization results of the NiO/C aerogel microspheres produced after the calcination in air: (**a**) low magnification image, (**b**) high magnification image, (**c**) cross-sectional image and (**d**) the corresponding EDS pattern.

After the calcination process of Ni/C aerogel in air, the sample preserved its spherical structure, as shown in Figure 3a, but the diameter of microspheres shrank severely to the average value of about 2.0 μm. The embedded particles were highly dispersed inside the carbon aerogel microsphere, exhibiting a plum-pudding structure, just like plums (particles) embedded in a pudding (carbon aerogel microsphere) matrix. The particles had submicron sizes ranging from 200 to 700 nm. Figure 3b is a magnified image of a broken microsphere with the internal particles exposed. It can be identified that the carbon aerogel microsphere had a mesoporous structure and contained some void spaces. The internal particles were submicron-sized spheres assembled by nanoparticles, quite different from the original nickel particles, which was caused by the oxidation. To observe the particles deep inside the carbon microsphere, the sample was ground for some time to produce a small number of fragments, and the resulting cross-sectional image is presented in Figure 3c. The particles showed the same morphology, and the EDS pattern recorded on the particle area (Figure 3d) showed only the peaks of Ni, O, and C, confirming that the internal particles were also oxidized.

**Figure 4:**
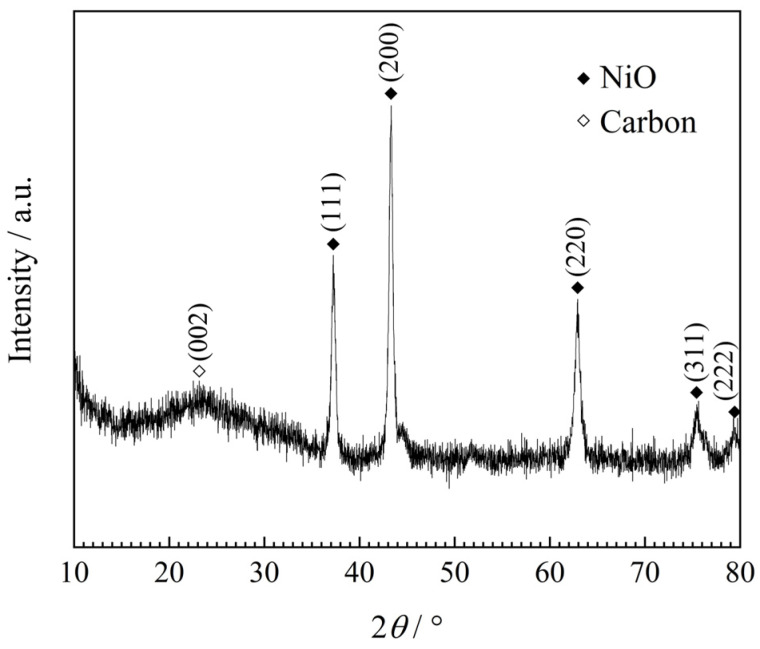
X-ray diffraction (XRD) pattern of NiO/C aerogel microspheres with a plum-pudding structure.

Figure 4 presents the XRD pattern of plum-pudding structured NiO/C aerogel microspheres. A group of strong diffraction peaks agreed well with the standard data of NiO with a rock-salt structure (JCPDS card no. 47-1049). The pattern also exhibited a broad peak around 23° with low intensity, which is the typical diffraction characteristic of amorphous carbon [29]. Diffraction peaks of other substances were not obvious, indicating that organic carbonization and nickel oxidation were both complete.

**Figure 5:**
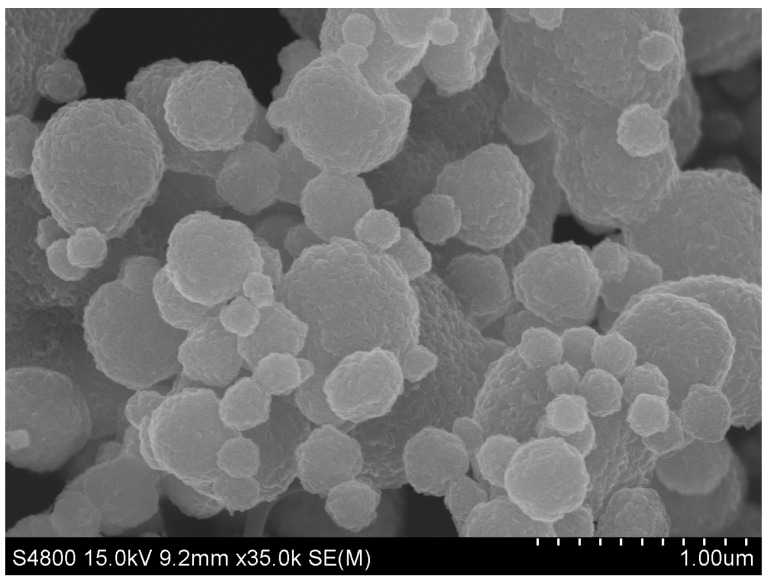
SEM image of NiO microspheres prepared by burning out the carbon aerogel in air.

Figure 5 shows the SEM image of the pure NiO sample produced by the calcination of Ni/C aerogel in air for 24 h. Apparently, the carbon aerogel was completely burnt out, leaving only NiO microspheres with a regular shape. According to the weight loss of the sample, it was calculated that the carbon content in the NiO/C aerogel composite was about 13 wt%.

**Figure 6:**
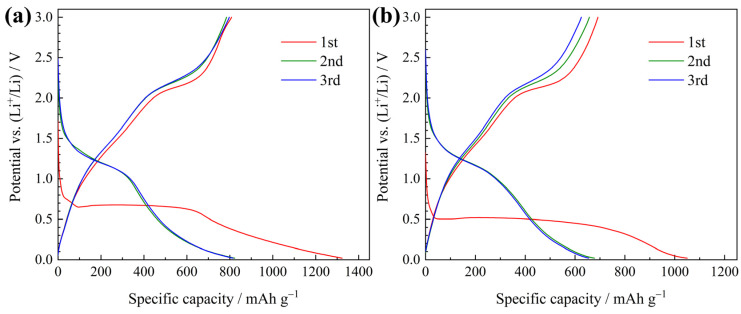
Galvanostatic discharge-charge curves of (**a**) the NiO/C electrode and (**b**) NiO electrode.

**Figure 7:**
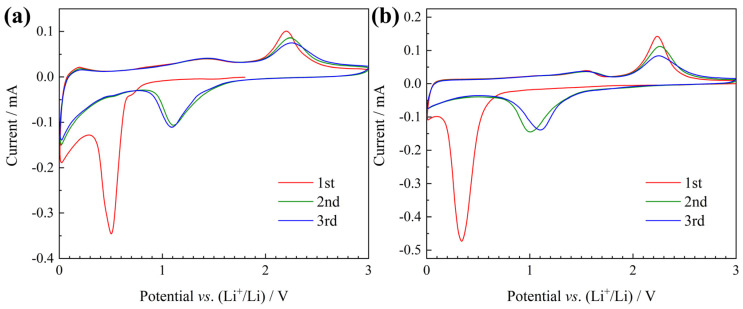
Cyclic voltammetry (CV) curves of (**a**) the NiO/C and (**b**) NiO electrodes.

As anode materials of lithium-ion batteries, the electrochemical performances of plum-pudding structured NiO/C microspheres and NiO microspheres were tested together for comparison. Their galvanostatic discharge-charge curves tested at 100 mA g^−1^ are compared in Figure 6. For both samples, except for the first irreversible discharge curve, the subsequent charge and discharge curves were similar. The NiO/C electrode delivers a first charge capacity (reversible capacity) of 808 mAh g^−1^, while for the NiO electrode, this value was 690 mAh g^−1^. The reversible capacity of the NiO/C composite exceeded the theoretical value of nickel oxide (718 mAh g^−1^), and the extra capacity should be ascribed from the carbon aerogel. According to the previously reported research, it is possible for the carbon aerogel to contribute a high reversible capacity derived from the micropores in the amorphous regions as well as the graphene layers in the graphite crystallites [29,30,31]. To study this further, the CV results are compared in Figure 7. Both electrodes showed a strong peak in the first cathodic curve, which corresponded to the first lithiation process including the formation of SEI and the conversion reaction of NiO to Ni. The subsequent anodic/cathodic curves were quite similar in shape, indicative of reversible reactions. The cathodic peaks around 1.1 V were related to the reduction of NiO to Ni and the anodic peaks around 2.2 V were attributed to the re-oxidation of Ni to NiO. However, there was a major difference between their curves. In the curves of the NiO/C electrode (Figure 7a), the CV characteristics of graphite (i.e. lithiation peak near 0 V and delithiation peak around 0.2 V) could be well distinguished, while in those of the NiO electrodes (Figure 7b), these peaks did not exist. This confirms the reversible lithium storage of the carbon aerogel.

**Figure 8:**
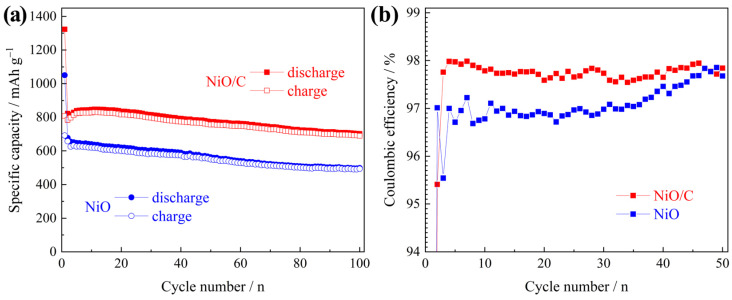
Comparison of (**a**) cycling performance and (**b**) coulombic efficiency between the two electrodes.

Figure 8a compares their capacity evolution until 100 cycles at a current density of 100 mA g^−1^. The NiO/C electrode showed a higher capacity throughout the cycle, and its reversible capacity retention after 100 cycles was 85%, higher than that of the NiO electrode (71%), and indicative of better cycling performance. Figure 8b compares the evolution of their coulombic efficiency. Apart from the first two cycles, the coulombic efficiency of NiO/C electrode was mostly higher, indicative of the better reversibility of electrochemical reactions.

**Figure 9:**
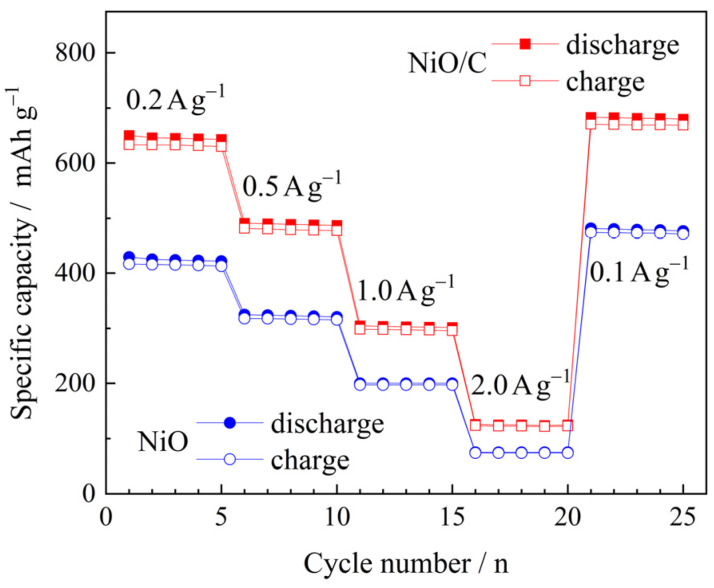
Comparison of the rate capability of two electrodes.

Figure 9 compares the rate capability of two electrodes. The cells, which have experienced 100 cycles at 100 mA g^−1^, continued to be tested at higher current densities of 200, 500, 1000, 2000 mA g^−1^, and eventually, 100 mA g^−1^. The capacities decreased with current densities, but at each current density, the NiO/C electrode delivered higher capacities than those of the NiO electrode, indicative of enhanced high-rate capability.

**Figure 10:**
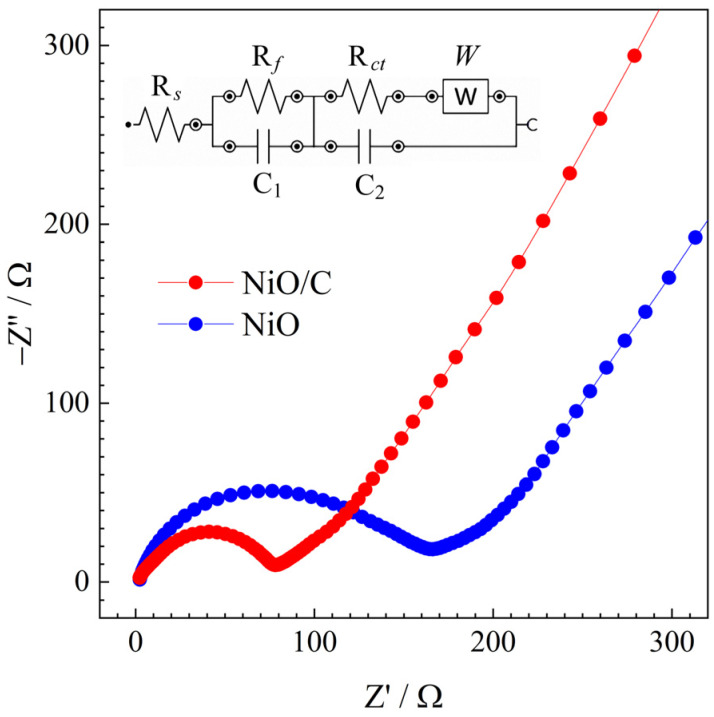
Comparison of the Nyquist plots of two electrodes.

Figure 10 shows the Nyquist plots of two electrodes after 100 cycles. The plots were composed of a semicircle in the medium frequency region and a sloping line in the low frequency region. The equivalent circuit inserted in the figure was used to fit the plot. The circuit components of R_s_, R_f_, R_ct_, W, and C represent the electrolyte resistance, SEI resistance, charge transfer impedance, diffusion-controlled Warburg impedance, and capacitance, respectively. The fitting results showed that the values of R_f_ and R_ct_ for the NiO/C electrode were 10.05 and 57.87 Ω, respectively, lower than those of the NiO electrodes (31.12 and 114.2 Ω, respectively), suggesting that the NiO/C electrode exhibited enhanced electrode kinetics.

The enhanced electrochemical performance of the NiO/C electrode was attributed to its plum-pudding nanocomposite structure, that is, spherical NiO particles were embedded in carbon aerogel microspheres. Carbon aerogel microspheres have at least four advantages. First, the carbon aerogel effectively disperses NiO particles, and enables each particle to fully participate in the electrochemical reaction. Second, the carbon aerogel can act as a porous elastic matrix, which can buffer the volume expansion of NiO particles and thus suppress pulverization. Third, the interconnected carbon microspheres can form a highly conductive network, which helps to improve electronic conductivity and reduce electrode polarization [32]. Fourth, the carbon aerogel is active toward lithium, which is beneficial to increase the capacity of the composite. As a result, the NiO/C electrode exhibits higher reversible capacity, better cycling stability, and high-rate capability.

Due to the above-mentioned structural advantages, relative to many previously reported NiO-based anode materials [15,17,33,34,35,36,37], the NiO/C aerogel microspheres with a plum-pudding structure in this present work also showed competitive electrochemical performance, as shown in Table 1. The enhancement of their electrochemical performance fully confirms the effectiveness of this structural design, and it is believed that this structure can also be applied to other electrode materials.

## 4. Conclusions

In summary, NiO/carbon aerogel microspheres with a plum-pudding structure were successfully prepared by a sol-gel process combined with a carbonization process in argon as well as a calcination process in air. The composite particle contained the carbon aerogel microspheres and the spherical NiO particles embedded inside. As anode materials for lithium-ion batteries, NiO/C aerogel microspheres exhibit significant enhancement in electrochemical performance including reversible capacity, cycling stability, and rate capability compared to those of the NiO microspheres. The enhancement is ascribed to the plum-pudding nanocomposite structure, as the carbon aerogel microspheres have the ability of stabilizing the structure of active materials, improving the electrode conductivity, and increasing the specific capacity.

## Figures and Tables

**Table 1:** Comparison in electrochemical performance between some published NiO-based anode materials and the NiO/C sample in the present work.

Material	Initial Reversible Capacity mAh g^−1^	Capacity Retention % (Nth)	Current Density mA g^−1^	Reference
NiO nano/microspheres	735	96 (100)	100	[15]
NiO/C hollow microspheres	760	83 (100)	100	[17]
Cu-doped NiO nanoflakes	1108.9	59 (50)	100	[33]
Ni−NiO/C nanocomposite	914.11	70 (300)	100	[34]
NiO/rGO nanoflowers	996.9	70 (100)	100	[35]
NiO double-shelled hollow spheres	964.3	14 (100)	200	[36]
NiO mesoporous nanorods	737	39 (100)	100	[37]
NiO/C aerogel microspheres	808	85 (100)	100	This work
